# Nuclear-Targeted Drug Delivery Systems for Cancer Therapy: Advances, and Challenges

**DOI:** 10.3390/molecules31091437

**Published:** 2026-04-26

**Authors:** Yonghui Liu, Yanan Wu, Yifan Wu, Dong Wan, Cheng Zhang, Yongzhen Pei, Jie Pan

**Affiliations:** 1 School of Chemistry, Tiangong University, Tianjin 300387, China; liuyonghui@tiangong.edu.cn (Y.L.);; 2 School of Chemical Engineering and Technology, Tiangong University, Tianjin 300387, China; 3 School of Mathematical Sciences, Tiangong University, Tianjin 300387, China

**Keywords:** nuclear-targeted, drug delivery system, tumor therapy, controlled release, anticancer

## Abstract

The development of nuclear-targeted drugs has provided a more favorable option for tumor therapy. However, multiple biological barriers in vivo greatly reduce the efficiency of drug entry into the nucleus, rendering tumor therapy largely ineffective. Notably, the nucleus remains a critical therapeutic target, as most anticancer agents exert their effects through direct interactions with nuclear DNA or inhibition of topoisomerase activity, thereby disrupting DNA structure and impeding replication/transcription processes. This review systematically examines advanced delivery strategies for nuclear-targeted drug systems, explores their diverse therapeutic applications in oncology, and analyzes current challenges alongside future opportunities to guide the development of next-generation intelligent nuclear delivery platforms.

## 1. Introduction

Cancer remains a major global health challenge, ranking as the second leading cause of death worldwide with escalating incidence and mortality rates [1,2,3,4,5]. Since tumor cells have the characteristic of evading immune surveillance and inhibiting the normal functioning of surrounding cells [6], they are resistant to being completely cured using conventional treatments. Molecularly targeted therapy, a precision drug delivery paradigm [7,8,9], addresses critical limitations of traditional approaches including poor specificity, suboptimal bioavailability, and systemic toxicity [10], while enabling site-specific accumulation for enhanced therapeutic precision. The advantage of this strategy lies in its capacity to deliver drugs directly and precisely to the target site, thereby improving the therapeutic efficacy and safety of the drug. In contrast, most conventional therapies suffer from poor efficacy because the amount of the drug that ultimately reaches and acts on the drug target is minimal. Therefore, more advanced drug delivery systems should be explored to deliver drugs to improve targeting efficiency and enhance cellular drug uptake. The nucleus is one of the most important organelles in the cell, which not only regulates essential cellular processes like growth and reproduction but also serves as the repository for genetic information [11]. The cell nucleus is regarded as an ideal target for various therapeutic agents, including drugs, genes, or proteins desired. In tumor therapy, the destruction of DNA or proteins in the nucleus is an effective means of inducing tumor cell death. Many chemotherapeutic agents, such as camptothecin, cisplatin, and doxorubicin (DOX) [12], target the nucleus as their ultimate target, and they induce cell death by interacting with DNA helices or related enzymes. Among them, camptothecin, which achieves its therapeutic purpose by inhibiting DNA replication and RNA synthesis, is a practical TOP I inhibitor [13]. Cisplatin, a DNA-binding agent, induces apoptosis by inhibiting cellular transcription and replication [14,15]. Furthermore, DOX is able to block DNA replication in the nucleus and induce apoptosis in tumor cells [16]. Numerous clinical studies have investigated DNA-damaging agents. Clinical data demonstrate that pharmacologically induced DNA damage is an effective strategy for tumor treatment. However, the associated systemic toxicities restrict their maximum tolerated doses [17,18].

**Scheme 1:**
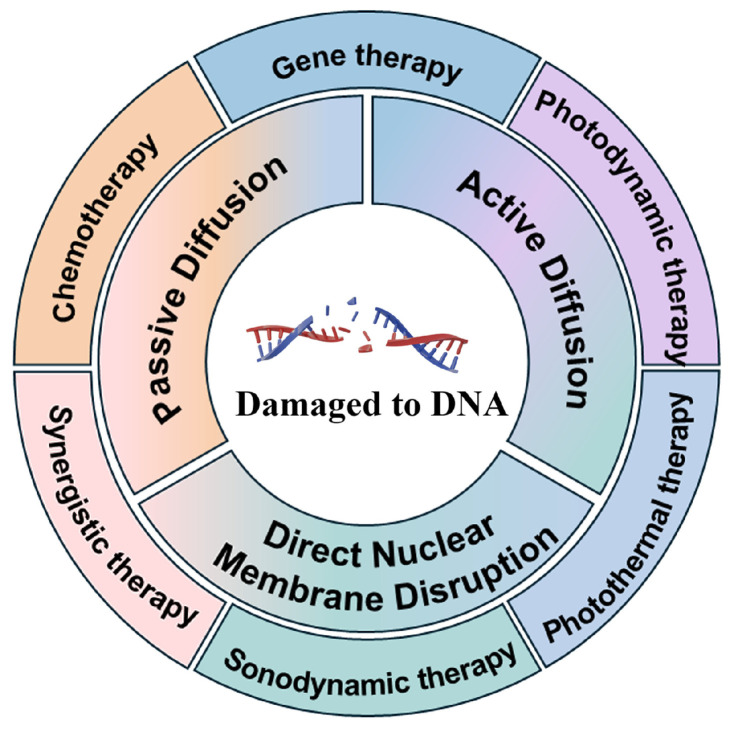
Illustration of nuclear-targeted drug delivery system for enhanced tumor treatments. This system can precisely target cancerous tissues, overcome barriers such as the tumor cell membrane, and ensure the accurate release of therapeutic agents within the cell nucleus, where they can exert their full therapeutic potential.

Consequently, developing high-performance nanocarriers capable of orchestrating relevant drug delivery to the cell nucleus represents a critical frontier in precision oncology. These intelligent systems effectively enhance the therapeutic effect, while minimizing the off-target toxicity of the encapsulated drugs by precisely targeting the tumor tissues, cells, and even organelles. This approach holds the potential to address drug resistance and ensure the safety and effectiveness of the treatment [19,20,21]. The nuclear-targeted drug delivery systems utilize advanced nanotechnology to process and modify the surface of the drug, progressively homing to tumor tissues, penetrating cellular membranes, escaping lysosomal degradation, and ultimately translocating through nuclear pore complexes (Scheme 1). Such multistage precision addresses two fundamental challenges: overcoming drug resistance through enhanced nuclear accumulation and ensuring treatment safety via minimized systemic exposure, as evidenced by recent advances in surface-engineered nanoplatforms.

Nevertheless, the engineering of nuclear-targeted delivery systems faces several significant challenges. To ensure effective delivery of the therapeutic drug into the nucleus, it is necessary to precede endosomal escape, cytoplasmic navigation, and ultimately nuclear envelope penetration—a cascade where over 90% of administered drugs typically fail. Upon systemic administration, these drugs must navigate a complex array of biological barriers within the circulatory system. The delivery efficiency is notoriously low; for every million nanoparticles administered, only approximately one is estimated to successfully reach the cell nucleus [22]. The cytoplasm is enveloped by filamentous cytoskeletal structures, which restrict the transport of macromolecules and nanoparticles; most macromolecules (such as HPMA copolymers and oligonucleotides) are primarily retained in endosomes/lysosomes following endocytosis, with an extremely low efficiency of escape into the cytoplasm [23].Consequently, escape from the endosomes/lysosomes into the cytoplasm must first be achieved before the macromolecules can interact with importin α/β via the nuclear localization signal (NLS) and be transported further to the perinuclear region or into the nucleus—this indicates that cytoplasmic delivery is the rate-limiting step in the entire nuclear-targeting process, whilst the cell nucleus serves as the ‘convergence point’ where macromolecules naturally tend to accumulate once they enter the cytoplasm. Coupling oligonucleotides with degradable linkers (such as GFLG) significantly enhances delivery efficiency to both the cytoplasm and the nucleus (escape begins after 2–4 h, with nuclear distribution observed in all cells by 24 h); whereas NLS modification alone, primarily due to increased positive charge enhancing membrane binding, has a limited effect on actual nuclear delivery. In contrast, Tat transmembrane peptide modification is the most efficient: it enables rapid entry into the cytoplasm via an energy-independent pathway within 5 min, with simultaneous nuclear entry; it remains effective even at 4 °C, completely overcoming the endosomal escape bottleneck [24]. Furthermore, there is a clear positive correlation between surface NLS density and the efficiency of nuclear/perinuclear accumulation; carboxyl groups remaining on the carrier surface and arginine/lysine residues within the NLS can promote endosomal membrane fusion, thereby enhancing cytoplasmic release; conversely, merely increasing positive charge (e.g., using randomized peptides) without NLS sequence specificity results in significantly lower nuclear-targeting efficiency. These results strongly demonstrate that enhancing nuclear-targeting efficiency requires first optimizing the cytoplasmic delivery (particularly endosomal escape) step, and that both NLS density and sequence specificity are indispensable. In addition, cancer cells deploy evolved resistance mechanisms including P-glycoprotein (P-gp) overexpression for drug efflux [25], enhanced drug metabolism [26], and increased lysosomal sequestration, among others. Such manners considerably reduce treatment efficacy and elevate the risk of chemotherapy failure. Multidrug resistance severely limits chemotherapeutic efficacy. Specifically, the P-gp-mediated active efflux depletes perinuclear drug concentrations, while upregulated DNA repair pathways (e.g., nucleotide excision and homologous recombination) counteract nucleus-acting agents like platinum drugs [27]. Additionally, densely packed heterochromatin restricts drug access to DNA compared to open euchromatin. Nucleus-targeted delivery systems can surmount these barriers via strategic design: utilizing NLS to bypass cytoplasmic P-gp, co-delivering PARP inhibitors to block repair machinery, and employing HDAC inhibitors to decondense chromatin for enhanced drug–DNA binding. Thus, these rationally designed systems present a potent strategy to circumvent conventional drug resistance [28,29]. Studies have shown that only a minute fraction of the administered drugs ultimately penetrate the nucleus to achieve their therapeutic objectives, with most chemotherapeutics undergoing premature inactivation before reaching their genomic targets. The tumor microenvironment is characterized by hypoxia, a dense extracellular matrix, tumor stromal cells, and elevated interstitial pressure. Specifically, hypoxia leads to poor blood supply in deep tissues and reduces endocytosis and nuclear transport capacity. A dense extracellular matrix forms a physical barrier that blocks or traps nanoparticles while compressing cells and impairing transport. Tumor stromal cells, such as cancer-associated fibroblasts, secrete matrix components, compress blood vessels, phagocytose particles, and downregulate nuclear transport proteins. Furthermore, elevated interstitial pressure counteracts convective transport, forcing particles to rely on slow diffusion and causing them to become trapped in the perivascular space. Ultimately, these four factors act synergistically to restrict nanoparticle penetration, induce stromal retention, and impede cellular uptake and nuclear import, rendering the delivery of anticancer drugs to their nuclear targets exceedingly difficult [30,31].

In general, this review synthesizes the design principles and fundamental techniques underlying nuclear-targeted nanocomposites that play an effective therapeutic role, and lists a bunch of strategies to build nanocarriers that help drugs enter tumor cells, making them work better, as shown in Table 1. It also provides insightful suggestions for the further construction of nanocomposites. The nanomedicine accumulates in tumor tissue after circulating through the bloodstream. By manipulating properties such as size, morphology, surface charge, and other factors, the drug is facilitated into the tumor cells. Following endocytosis, nanocarriers are trafficked through the endocytic pathway and must undergo endosomal escape to release their cargo into the cytoplasm. This escape is a critical rate-limiting step; if nanocarriers remain trapped within endosomes or lysosomes for an extended period, they will be degraded by the acidic environment and hydrolytic enzymes. Currently, several endosomal escape mechanisms have been exploited in nanocarrier design. The most classic mechanism for cationic polymers, such as polyethyleneimine and polyhistidine, is the “proton sponge” effect. As the endosome acidifies, polymers with abundant protonatable groups continuously sequester protons, triggering an influx of chloride ions and water. This leads to osmotic swelling and eventual rupture of the endosomal membrane. Alternatively, certain peptides and proteins undergo conformational transitions in the acidic endosomal milieu, forming transient pores to facilitate escape. Photochemical internalization offers spatiotemporal control by co-delivering a photosensitizer; upon irradiation at a specific wavelength, the generated reactive oxygen species selectively disrupt the endosomal membrane, thereby reducing off-target toxicity. Finally, pH-sensitive chemical bond cleavage utilizes nanogels or polymers incorporating acid-labile bonds. Under endosomal acidic conditions, the carrier structure collapses or its surface charge reverses (e.g., from negative to positive), promoting membrane interaction and inducing escape [32,33,34].

Subsequently, the therapeutic efficacy of the drug is optimized by promoting its entry into the cell nucleus via either active or passive targeting mechanisms. Once the drug concentration released at the site of action reaches a therapeutic threshold, it induces apoptosis in tumor cells, thereby accomplishing the objective of cancer treatment. Finally, the review provides insights and recommendations on the current challenges associated with nuclear-targeted nanocomposites and their future directions, with the aim of improving therapeutic efficacy, reducing side effects, and accelerating their clinical translation.

## 2. Strategies for Constructing Nuclear-Targeted Vectors

The nuclear membrane serves to separate the nucleus from the cytoplasm, while the nuclear pore complexes embedded in the membrane facilitate bidirectional communication between the cytoplasmic and nucleoplasmic compartments. Numerous nuclear pore proteins, generally composed of disordered phenylalanine-glycine repeats [63], are assembled in the inner wall of the nuclear pore complex, and their presence provides selectivity for the entry of substances into the nucleus. Previous studies have demonstrated that molecules or particles with molecular weights below 40 kDa can freely enter and exit the nucleus through NPC, whereas relatively large substances are excluded from entering the nucleus by the phenylalanine-glycine nuclear pore proteins. This molecular sieving mechanism necessitates three strategic approaches for nuclear drug delivery: passive diffusion for small-molecule therapeutics, NLS-mediated active transport for macromolecules, and transient nuclear envelope disruption. Notably, NLS conjugation to nanocarriers enables receptor-mediated NPC traversal, and a range of drugs capable of being effectively targeted to the nucleus has been developed.

### 2.1. Passive Diffusion

Passive nuclear entry operates through Brownian-driven stochastic motion, where no energy is consumed during the passive diffusion of small molecules into the nucleus. This represents the primary pathway through which small molecules that are sub-40 kDa enter the cell nucleus. It has been demonstrated that nanoparticle diffusion dynamics are governed by structural determinants, the primary factors influencing the passive diffusion of nanoparticles are their size and shape.

#### 2.1.1. Size Control

**Figure 1:**
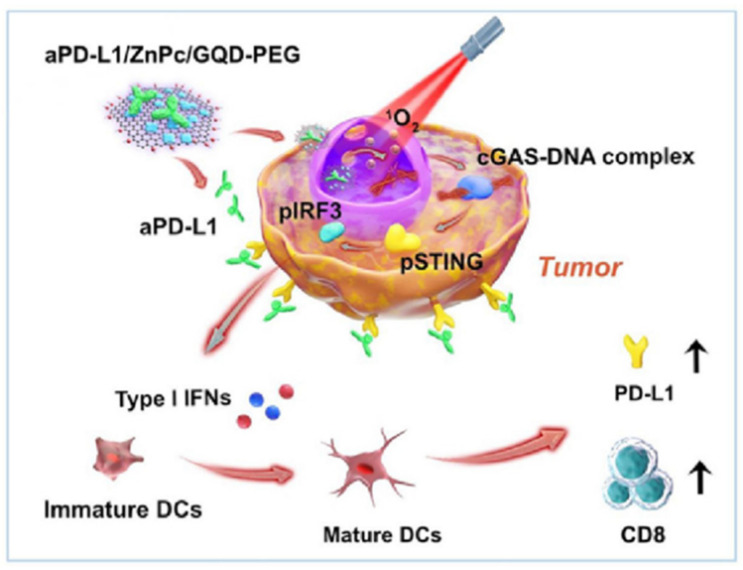
Schematic illustration of the nuclear-located PZGE nanoparticles for in situ cGAS/STING/IFN I innate immune signaling pathway activation-enhanced “cold tumor” photoimmunotherapy. Reprinted with permission from [35]. Copyright 2023 Elsevier.

To investigate the maximum size of nanoparticles that can freely enter the nucleus, Liang et al. conducted an experiment on the penetration of NPCs by nanoparticles of different sizes [64]. Based on their research into the maximum particle size capable of entering the cell nucleus via free diffusion, they designed a pair of Au nanoparticles with four different particle sizes (2, 6, 10, 16 nm). By observing the Bio-TEM results of MCF-7 cells treated with different sizes of Au NPs for 24 h, it was found that a sharp 9 nm cutoff—particles below this threshold (2–6 nm) achieved nuclear localization, while larger counterparts (≥10 nm) remained cytoplasmically restricted. This seminal finding established the <9 nm “nuclear gatekeeping” rule. Applying this result, Zhang et al. used graphene quantum dots (GQDs) as a carrier for aPD-L1 and zinc phthalocyanine (ZnPc) [35] and ultimately designed 5 nm aPD-L1/ZnPc/GQD-PEG (PZGE) nanoparticles with excellent enrichment capabilities (Figure 1). PZGE-based photodynamic therapy can enhance type I IFN-mediated innate immune responses and transform “cold tumors” into “hot tumors”. Taking advantage of their size to target the nucleus, the 5 nm nanoparticles induce DNA damage and activate the type I IFN-mediated innate immune response, thereby promoting cytotoxic T-lymphocyte infiltration and reversing negative PD-L1 expression.

#### 2.1.2. Shape Control

**Figure 2:**
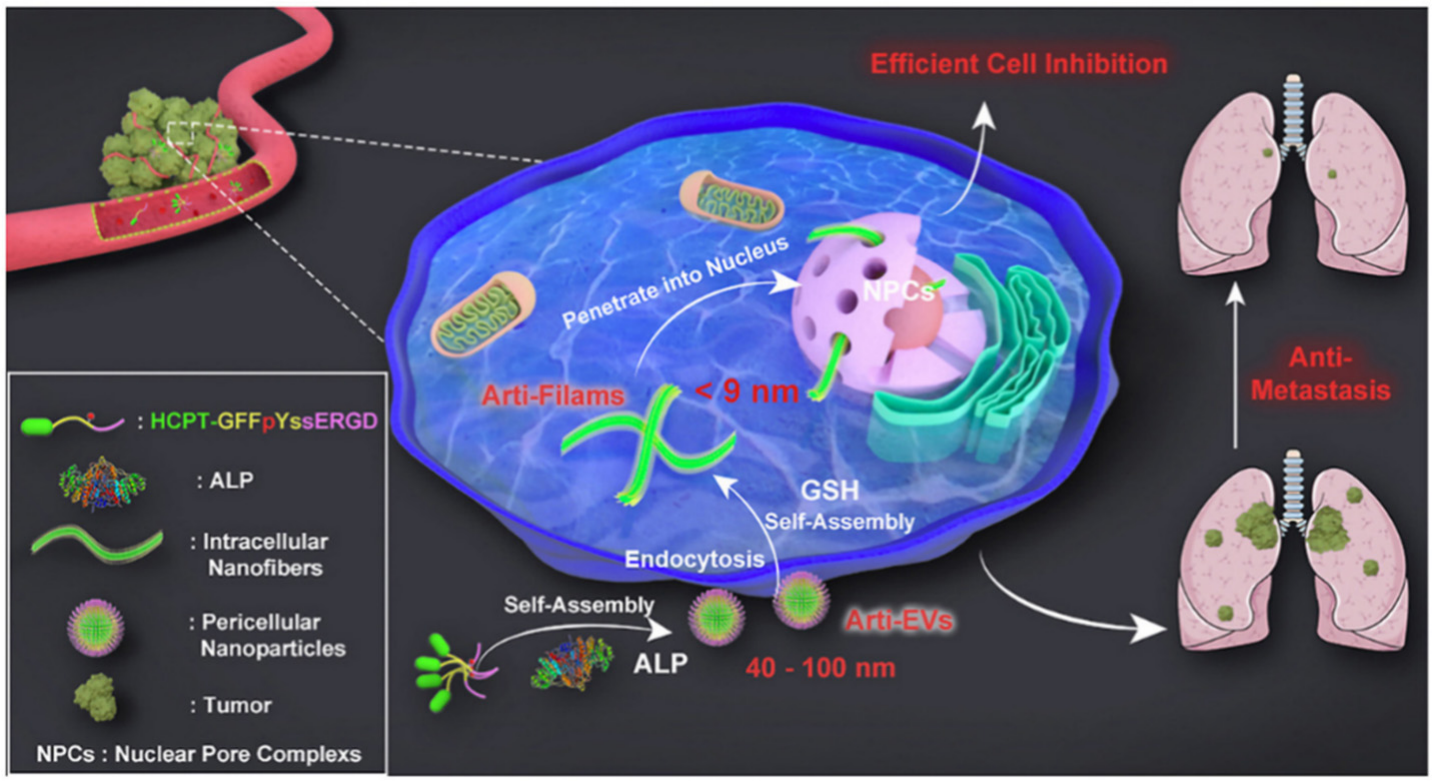
The nuclear–targeting delivery, based on an organelle-mimicking cascade delivery strategy, thereby effectively suppressing liver cancer and lung metastasis. Reprinted with permission from [65]. Copyright 2022 Elsevier.

Regarding the effect of nuclear diffusion of nanoparticles with different shapes, Gaus and his colleagues designed four block copolymers with the same surface properties but different geometries, and the article presents a detailed study of the cellular and subcellular behavior of these four nanoparticles [36]. It was found that among rod-like (5–10 nm × 100–300 nm), worm-like (5–10 nm × 400–700 nm), ring-like, and spherical nanoparticles, those with elongated high-aspect-ratio architectures (rod and worm variants) exhibited superior nuclear membrane penetrability. After encapsulating doxorubicin (DOX), the rod- and worm-shaped particles demonstrated significantly enhanced delivery of dox into the nucleus, resulting in significantly higher dox concentrations than the other two due to the nuclear diffusivity of the spherical shape. Zhang and his team engineered a stimuli-responsive nano platform HpYss mimicking organelle-level adaptability for precision oncology. It consisted of the 10-hydroxycamptothecin (HCPT) peptide coupler, which can emulate the extracellular vesicle-to-fiber transition, and dynamically adjust its structure and function in response to intracellular environmental changes for precise liver cancer treatment(Figure 2) [65]. Under the successive stimulation of extracellular alkaline phosphatase (ALP) and intracellular glutathione (GSH), HpYss undergoes a morphological transformation from spherical nanoparticles (50–100 nm) to rod-like nanofibrils (4–9 nm) through tandem self-assembly. HpYss exhibits exceptional nuclear enrichment capabilities within HepG2 cells, potentiating the cytotoxic effects of HCPT. Moreover, HpYss effectively penetrates tumor tissue, achieving high accumulation and prolonged retention within the tumor, leading to significant suppression of tumor growth and prevention of lung metastasis.

### 2.2. Active Diffusion

While passive diffusion enables nuclear entry of ultrasmall nanoparticles, this mechanism imposes critical constraints on both drug payload capacity and delivery kinetics due to strict size-dependent permeability. To transcend these limitations, engineered active nuclear–targeting strategies have emerged as essential tools for nanocarrier design. For larger nanocomposites (50–200 nm), precise nuclear localization is achieved through two synergistic approaches: surface functionalization with NLS peptides to exploit endogenous transport machinery, and charge-mediated nuclear envelope penetration via zwitterionic surface engineering.

#### 2.2.1. Ligand Modification

Nuclear-targeting of particles exceeding the size of NPC can be achieved by attaching a nuclear localization signal, NLS, on the particle surface, which is used to overcome the size limitation of NPC for nano-delivery composites. These particles enter the nucleus against the concentration gradient via active transport mediated by carrier proteins, which dissipate energy.

**Figure 3:**
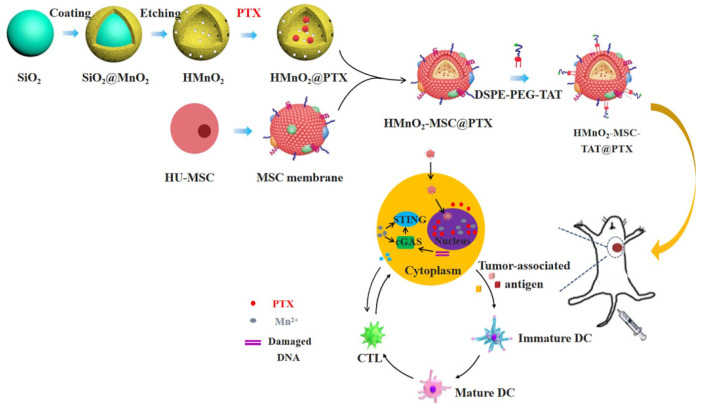
The preparation of HMnO_2_-MSC-TAT NPs and the way to realize chemo-immunotherapy. Reprinted with permission from [38]. Copyright 2023 American Chemical Society.

The TAT peptide is a transcriptional activator [66], rich in arginine and lysine. It primarily modulates the efficient and sequential transcription of long terminal repeat (LTR) sequences in human immunodeficiency virus type 1 (HIV-1) by binding to input proteins, facilitating entry into the nuclear pore [67,68]. The TAT peptide is one of the most commonly used NLSs. Nowadays, a multifunctional nano drug delivery system (NDDS), which can accomplish surface charge inversion and nuclear-targeting, has been proposed by Jing et al. [37]. This NDDS utilized a poly(ethylene glycol)-poly(ε-caprolactone) copolymer modified with 2,3-dimethylmaleic anhydride and TAT peptide (PECL/DA-TAT), where DA concealed the positive charge of the TAT to extend drug circulation in vivo. When the material accumulated approaching acidic tumor tissue, internalization was facilitated by a pH-triggered change in the negative to the positive charge on the surface of the NDDS. Subsequently, the drug entered the nucleus mediated by the TAT peptide. Xie and his colleagues designed a smart biodegradable hollow manganese dioxide (HMnO_2_) nanoparticle with a membrane coating of human umbilical cord mesenchymal stem cells (HUC-MSC) and modified it with TAT peptide to produce HMnO_2_-MSC-TAT@PTX material (Figure 3) [38]. This material exhibits the active targeting ability of HUC-MSCs, which can show significant tumor aggregation at the cancer site, and TAT modification enables active nuclear-targeting of drugs. Moreover, this material effectively promotes the maturation of dendritic cells and activates effector T cells to act at the tumor site.

Organic boron compounds are a crucial class in organic synthesis, demonstrating versatile reactivity that enables diverse applications in chemical synthesis. Notably, phenylboronic acid (PBA) exhibits unique dynamic covalent behavior through its reactions with 1,2- and 1,3-diols, forming reversible boronic acid ester bonds that have been extensively exploited in biomedical systems [69,70]. This dynamic binding capability is particularly valuable for tumor-targeting applications, as PBA demonstrates a high association constant with salivary acid—a carbohydrate overexpressed on cancer cell surfaces—under weakly acidic conditions typical of tumor microenvironments [71,72,73]. Furthermore, the cellular internalization of boronic esters is facilitated by active transport mechanisms, primarily via the importin α/β nuclear transport pathway, enhancing their potential as drug delivery vectors. Liu and his colleagues designed a polydopamine (PDA) nanoparticle that modified polyethylene glycol (PEG) on the surface of PBA by dynamic interactions [39]. Utilizing the cleavable properties of borate bonds in acidic environments, the PEG coating is effectively removed within the tumor microenvironment, which in turn exposes PBA groups to target the nucleus, allowing precise release of phototherapeutic or chemotherapeutic agents into the nucleus. Tang et al. used PBA to deliver proteins precisely into the nucleus, solving the problem that the macromolecules are unable to load with traditional passive targeting [40]. The results show that the boronic esters serve as an effective mediator for the active intranuclear transport of proteins, resulting in precise nuclear localization and enhanced utilization of macromolecular cargoes. This strategy of targeting the nucleus using borate provided a novel, fully synthetic mode of drug delivery that is minimally intrusive and can be engineered into permanent or cleavable forms as needed for greater flexibility.

Therapeutic peptides have emerged as pivotal agents in targeted drug development and cancer therapy, owing to their exceptional target specificity, molecular selectivity, and favorable biocompatibility profiles [74]. Among these, cell-penetrating peptides (CPPs) are a distinctive subclass that facilitates the intracellular delivery of diverse cargoes that mediate the passage of viruses, plasmid DNA, siRNAs, and other small molecules across the nuclear plasma membrane [75]. The TAT peptide previously described is one of them. CPPs are systematically categorized into three mechanistic classes based on their physicochemical properties: cationic, hydrophobic, and amphiphilic peptides [76]. Cationic peptides, as the name suggests, are characterized by abundant arginine or lysine residues which are able to form the -NH3+, and demonstrate remarkable cellular uptake efficiency; and experiences have shown that small sequences containing just eight arginine residues can be effectively taken up by cells [77]. Hydrophobic peptides are derived from signaling peptides, and transporter proteins are one type of hydrophobic peptide. Tryptophan residues in hydrophobic peptides play an important role in the interaction with membranes [74]. Compared to the other two classes of CPPs, hydrophobic CPPs are less investigated [78]. Amphiphilic CPPs contain hydrophobic structural domains that can undergo fusion with the NLS [74]. Amphiphilic CPPs can be classified into primary amphiphilic CPPs and secondary amphiphilic CPPs. Amphiphilic CPPs utilize hydrophobic domain membrane anchoring coupled with hydrophilic domain solvation for cellular entry [66], while secondary amphiphilic CPPs adopt α-helical conformations that spatially segregate hydrophobicity and charge. By means of a helical wheel, this structure allows the peptide sequence to accumulate a high concentration of hydrophobic residues on one side and cationic, anionic, or polar residues on the other, thus exhibiting a unique amphiphilic property [79].

Metal-based therapeutics achieve DNA targeting through covalent coordination with nucleobase heteroatoms [80], a strategy particularly effective for disrupting genetic integrity. The polyanionic nature of DNA backbone phosphate groups drives electrostatic attraction with cationic metal complexes, facilitating the formation of DNA adducts that induce structural distortions and replication errors, which are the key mechanisms underlying their genotoxic effects [81,82]. Beyond covalent interactions, there are three other reversible modes of non-covalent binding, which are groove binding, embedding and insertion [83]. Groove binding exploits shape complementarity through hydrogen bonding and van der Waals interactions within DNA helical grooves, distinct from π-stacking mechanisms. This binding mode not only stabilizes DNA-small molecule complexes but also synergizes with chemotherapeutic agents to induce cell cycle arrest through double-strand break formation [84]. Intercalative complexes employ planar aromatic ligands that insert between base pairs, with metal coordination centers precisely positioned to recognize specific nucleotide sequences. The preorganized three-dimensional architectures of these complexes enable both structural recognition and redox-mediated DNA damage via Fenton-type reactions [85]. Gao et al. have designed and synthesized a nanocluster containing copper ions [86], using this artificial metalloenzyme designed as an active center to react temporarily with substrates to form intermediates that provide lower energy transition states, thereby accelerating the chemical reaction. This material selectively binds to DNA in cancer cells and catalyzes the DNA breakage reaction, leading to the death of the cancer cells.

#### 2.2.2. Charge Reversal

**Figure 4:**
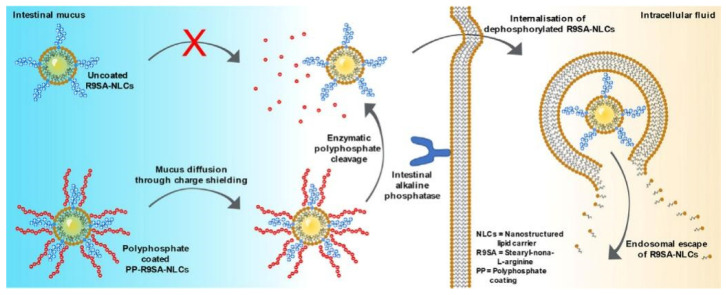
Schematic illustration of the PP-R9SA-NLCs, coating with PP can achieve charge conversion at target cells. Reprinted with permission from [78]. Copyright 2022 Elsevier.

The effect of surface charge of Au NPs on the intracellular accumulation in human fibroblasts (1BR3G) by Puntes et al. evidenced that cationic surface modifications significantly enhance nuclear membrane penetration compared to neutral or anionic counterparts [41]. Therefore, the concentration of drugs at the targeting site can be enhanced by achieving a reversible surface charge of nanoparticles at the target site, transitioning from negative or neutral to positive. The charge reversal of smart nanoplatforms demonstrated enhanced drug accumulation at tumor sites through microenvironment-triggered (e.g., light, heat, enzymes, and pH changes) transformations [87]. They prolonged circulation via stealth properties in neutral physiological environments, then strengthened tumor cell adhesion and promoted cellular internalization via charge-mediated endocytic pathways. As Figure 4 shows, Knoll et al. developed a nanostructured lipid carrier (NLC) modified with polycationic CPP, and polyphosphate (PP) coating is used to mask that positive charge [88]. It can contribute to triggering charge reversing in target cells via intestinal alkaline phosphatase (IAP), a membrane-bound enzyme. This material, a PP-stearoylamino-l-arginine (R9SA), was prepared by solid-phase synthesis. It improved the mucus permeability of the drug and increased the cellular uptake of the drug simultaneously. The PP layer masked the positive charge on the surface of R9SA, and when the material was co-colonized with alkaline phosphatase and Caco-2 cells, the surface charge of the nanocarrier switched to allow higher cellular uptake by the Caco-2 cells, followed by the drug being guided by the cations on the surface into the nucleus [42]. The emergence of this material has driven the development of mucosal drug delivery nanocarriers.

### 2.3. Direct Nuclear Membrane Disruption

**Figure 5:**
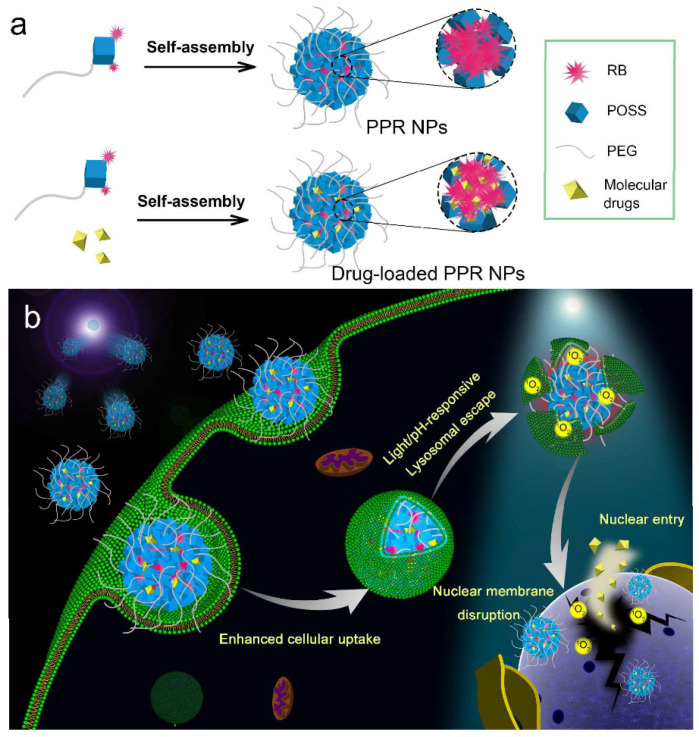
(**a**) Schematic illustrating the formation of PPR NPs and PPR/HCPT NPs. (**b**) Schematic illustration of the pH-responsive and light-triggerable nuclear delivery strategy using PPR/HCPT NPs as an example. Reprinted with permission from [44]. Copyright 2018 American Chemical Society.

The ultimate challenge in delivering a drug to the nucleus is letting it cross the nuclear membrane via the NPC; however, the nuclear membrane poses a significant barrier to the entry of nuclear-targeted drugs due to the restrictive size of the nuclear pores. The above detailed the modification of nuclear-targeted nanocarriers for NPC recognition and cytoplasmic-to-nuclear shuttling via NLS modifications [89,90,91,92,93]. This section will describe the adjustment of NPCs. Enlargement of NPCs by pharmacologically induced, dramatically enhancing nuclear membrane permeability for therapeutic cargos. Winnok H. De Vos et al. devised a technique based on vapor nanobubble(VNB)-mediated light-induced disruption of the nuclear envelope (NE) [43]. Irradiation of gold nanoparticles around the nucleus by a wide-field laser led to the formation of transient vapor nanobubbles, which caused minor mechanical damage to the NE, resulting in the creation of small pores. Gold nanoparticles (AuNPs) were harnessed to enhance the efficiency and throughput of cells to drugs. Their experiments demonstrated that 70 kDa and 150 kDa FITC dextran entered into the nucleus following nuclear photodegradation, with the estimated diameters of these molecules being 11.1 nm and 16.6 nm, respectively. Application of this result culminated in the transport of gold nanoparticles into the cell nucleus, where spontaneous nuclear rupture occurs. The construction of a universal nuclear-targeting system was reported by Zhu et al. [44]. As Figure 5 shows, this system, nanoparticulate PPR NPs, was self-assembled by a photosensitizer rose Bengal (RB), hydrophilic PEG, and polyhedral oligomeric silsesquioxane (POSS). Once irradiated by mild laser, the PPR NPs generate reactive oxygen species, which effectively disrupt the lysosomal structure it adheres to bind in high concentrations on the nuclear membrane, further disrupting the integrity of the nuclear membrane and delivering the loaded chemotherapeutic drug into the nucleus. The construction of this nuclear-targeting system effectively avoids the complex modification of nuclear-targeting ligands and is generally applicable to molecular compounds and nanomaterials.

## 3. The Application of Nuclear-Targeting Nanomaterials in Tumor Treatment

Applying the above principles to the construction of nuclear-targeted nanomaterials, this chapter will discuss how these principles can be applied to tumor therapy.

### 3.1. Chemotherapy

The nucleus is a critical organelle in the cell; it serves as the ultimate therapeutic battlefield for many chemotherapeutics. Various chemotherapeutic agents exert their therapeutic effects within this compartment, including drugs like doxorubicin (DOX), cisplatin, and carboplatin. These agents require nuclear access to induce DNA damage or disrupt replication machinery, so the effectiveness of chemotherapy is fundamentally governed by their nuclear accumulation kinetics. Low doses of traditional chemotherapeutic agents could reach intranuclear targets due to multilayered biological barriers, and tumor cells exacerbate this challenge through adaptive resistance mechanisms. To address these biological defenses, a variety of nuclear-targeted NDDS have emerged to enhance the efficiency of free drug entry into the nucleus. Compared to traditional nuclear localization strategies for small molecule drugs, nuclear-targeted drug delivery systems exhibit higher bioavailability, greater stability, and enhanced nuclear-targeting ability, reducing non-specific drug distribution in vivo and lowering drug dosage.

**Figure 6:**
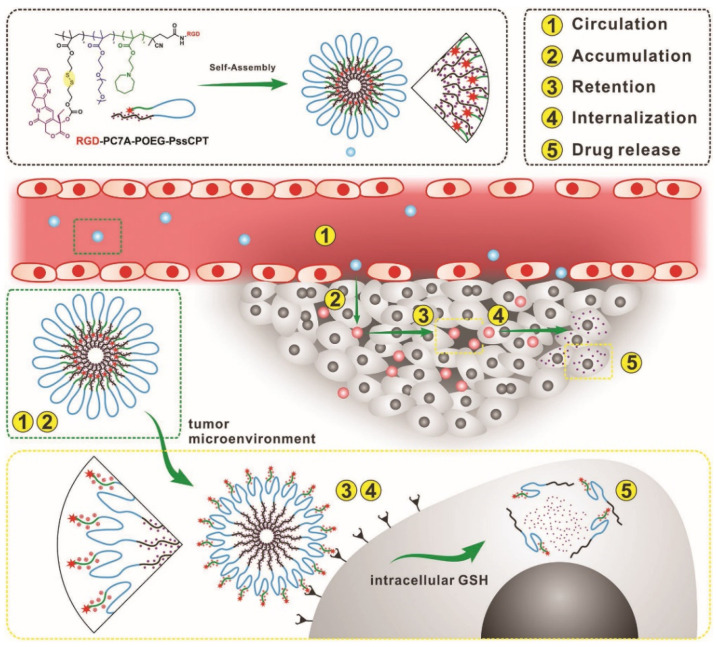
Schematic illustration of HRNMs for programmed CARIR chemotherapeutic delivery. The HRNMs show high stability in blood circulation. Reprinted with permission from [45]. Copyright 2018 Wiley.

Wang et al. designed a hierarchical responsive nanomedicine(HRNM), which self-assembles of poly(2-(hexamethyleneimino)ethyl methacrylate)-poly(oligo- (ethylene glycol) monomethyl ether methacrylate)-poly[reduction-responsive camptothecin] (PC7A-POEG-PssCPT) and cyclic Arg-Gly-Asp (RGD) peptide conjugated triblock copolymer, the scheme of which was described in Figure 6 [45]. The peptide RGD can be masked by the POEG coating to achieve long circulation in the bloodstream. As this HRNM enters the tumor tissue through transcytosis, the acidic tumor microenvironment triggers a charge reversal in the PC7A chain, which will expose the RGD peptide. This exposure enhances the retention of HRNMs at the tumor site and promotes their interaction with negatively charged cell membranes, which will increase intracellular delivery. Eventually, in the presence of GSH, the positively charged CPT enters the nucleus and attains the purpose of chemotherapy.

Zhang et al. designed and fabricated nuclear-targeted nanomaterials of LMHP-DOX mediated by histone 1 (H1), formed by bonding low-molecular-weight heparin (LMHP) to DOX and self-assembled to form LMHP-DOX NPs [46]. H1 binds to the cell membrane and is instrumental in establishing and preserving higher-order chromatin structures. Highly expressed H1 was present in human prostate cancer PC-3M cells. The prepared LMHP-DOX NPs were taken up by PC-3M cells and localized in the perinuclear region after a lysosomal escape, which mediated the entry of DOX into the nucleus of the cells through H1, rapidly increasing the concentration of DOX in their nucleus, thus enhancing the apoptotic activity of the targeted cancer cells.

**Figure 7:**
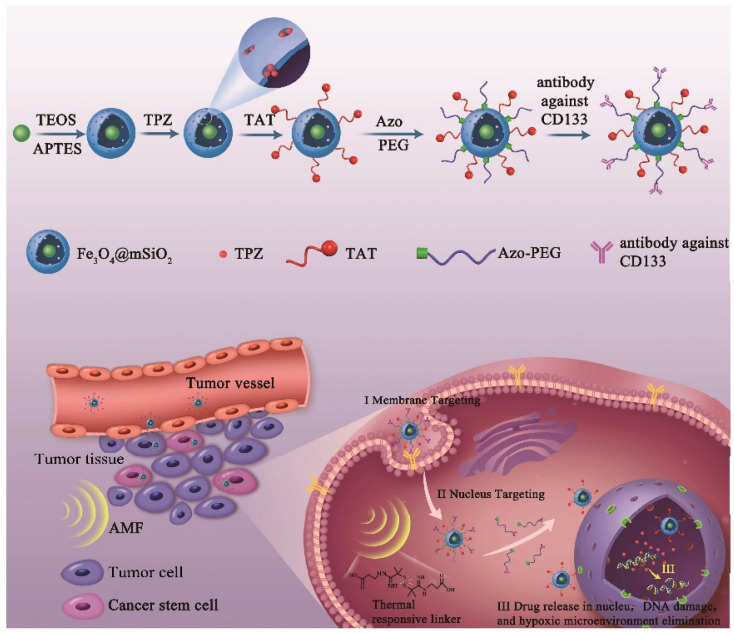
Fabrication of antibody against CD133/TAT/TPZ-Fe_3_O_4_@mSiO_2_ NPs, along with a diagram illustrating the multistage targeting strategy for cancer stem cell therapy. Reprinted with permission from [47]. Copyright 2019 Elsevier.

Li and his team engineered a mesoporous silica nanoparticle (MSN)-based nuclear-targeting system, CD133/TAT/TPZ-Fe_3_O_4_@SiO2, capable of directly targeting cancer stem cells (CSCs), as depicted in Figure 7 [47]. This system gains entry into the nucleus through a dual mechanism involving anti-CD133 surface modification for CSC recognition and the thermally induced exposure of TAT peptides under an alternating magnetic field (AMF). Nuclear-targeted drug release ultimately led to complete apoptosis of CSCs through combined hyperthermia and hypoxia-activated chemotherapy. The surface-modified anti-CD133 antibody directs the material to target the tumor tissue, thereby enhancing drug uptake by cancer stem cells (CSCs) and their differentiated progeny. In the presence of AMF, the breaking of the azo bond in the material exposes the TAT peptide to CSCs-nuclear targets. Eventually, the MSN encapsulating the antitumor drug tirazamine (TPZ) was mediated by the TAT peptide into the nucleus. This system effectively eliminated CSCs by blocking the hypoxia signaling pathway and is expected to provide new insights into the development of an efficient platform for CSC-targeted cancer therapy.

Xiang and his colleagues facilitated a novel one-dimensional semiconducting zinc coordination polymer (Zn-QCDM) [48], which can achieve selective targeting of nucleolus by Zn-QCDM through the action of the unpaired bases on RNA with zinc ligand with strong 7C-7C stacking and Zn-O coordination. When fitted with the chemotherapeutic drug camptothecin (CPT), Zn-QCDM can effectively deliver the drug to the nucleus of the cell, thus significantly enhancing the therapeutic effect.

### 3.2. Gene Therapy

Tumor gene therapy is a biotechnological approach that involves the insertion of therapeutic genes into host cells to suppress or eliminate the expression of oncogenes, thus achieving the complete eradication of the disease [13]. The nucleus functions as the repository of genetic information in the cell, and the effectiveness of gene therapy depends on the ability of the powerful but fragile therapeutic genes to be delivered to the nucleus [94,95], preserving their integrity and preventing degradation by cellular enzymes [13].

**Figure 8:**
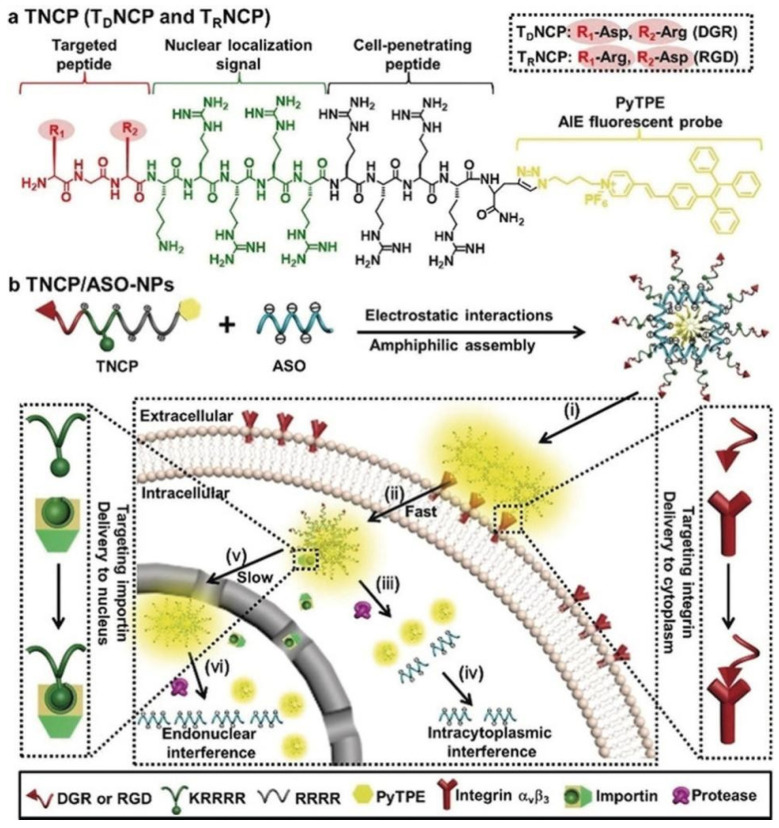
Schematic illustration of TNCP/ASO-NPs. (**a**) Molecular structure of TNCP. (**b**) Utilization of TNCP/ASO-NPs for real-time tracking of targeted delivery to the nucleus: (i) Specific binding to integrin αvβ3 receptors. (ii) Swift entry into the cytoplasm. (iii) Partial degradation by proteases and disassembly within the cytoplasm. (iv) Partial release of antisense oligonucleotides (ASO) in the cytoplasm, leading to intracellular interference. (v) Selective interaction with importins for nuclear import. (vi) Liberation of the majority of ASO within the nucleus, resulting in endonuclease interference. Reprinted with permission from [49]. Copyright 2019 Wiley.

Cheng and his associates have proposed a universal gene delivery strategy through the creation of an integrin-targeted, cell-penetrating, and nucleoplasmic transport peptide-conjugated aggregation-induced emission luminogens (AIEgen) called TDNCP, a material that enables targeted delivery of antisense single-stranded DNA oligonucleotides (ASOs) to the cell nucleus (Figure 8) [49]. Compared with TDNCP/siRNA-NPs (siRNAs act mainly in the cytoplasm), TDNCP/ASO-NPs (ASOs act mainly in the nucleus) showed better interfering effects, further suggesting that TDNCP is a nuclear-targeting carrier. TDNCP consists of four main components:(1) The RGD or DGR targeting peptide is capable of specifically binding to the αvβ3 integrin receptor, which has high expression in tumor cells and increases drug uptake by tumor cells. (2) The nuclear localization signal KRRRR, facilitating its operation within the nucleoplasm. (3) The cell-penetrating peptide RRRR, composed of positively charged amino acids, significantly enhances the ability to transport substances into the cell and nucleus, and also increases the ability of endosomes to escape. At the same time, it can be combined with therapeutic genes by electrostatic action, which improves the loading efficiency and specificity. (4) PyTPE, which is an aggregation-induced emission (AIE)-based molecule, is an azide-functionalized tetraphenylethylene derivative with cellular imaging fluorescence properties, allowing real-time monitoring of the material’s activity in the cell. At last, Western blot experiments showed that TDNCP/ASO-NPs effectively inhibited the expression of Bcl-2.

**Figure 9:**
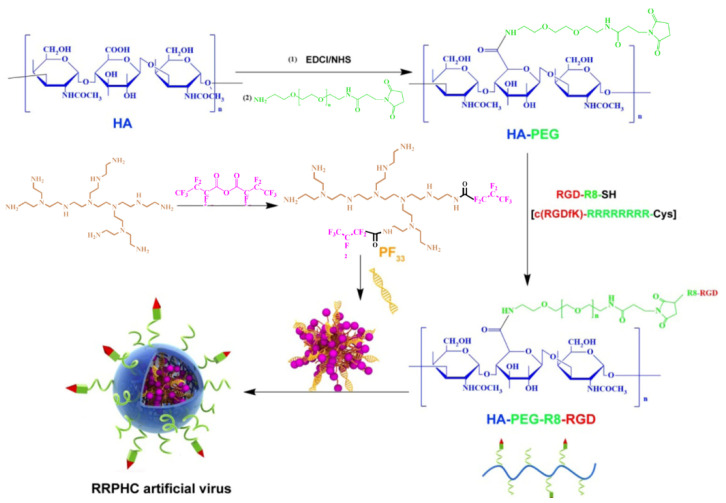
Schematic representation of RRPHC nanoparticles for gene delivery. Reprinted with permission from [50]. Copyright 2017 Ivyspring International.

Li et al. developed a multifunctional nuclear-targeted gene NDDS, RRPHC/hTRAIL, which has the ability to specifically bind CD44 (a key marker for cancer stem cell-like cells subpopulations) with HA, thereby precisely targeting and removing tumor stem cell-like cells, providing a new avenue for the treatment of rectal cancer (Figure 9) [50]. 1.8K PEI (PF33) modified with heptafluorobutyric acid was mixed with plasmid to form dense nanoparticles PFC (Fluorinated PEI/plasmid Complex). Modification of hyaluronic acid (HA) with peptides, attached with polyethylene glycol, constitutes RGD-R8-PEG-HA (RRPH), which is coated on PFC to finally constitute RRRPHC. It is able to achieve long-term recycling and penetration into tumor tissues while maintaining the high transfection efficiency of PFC. The in vivo distribution map showed that RRRPHC could achieve accumulation in the tumor region. In this system, TRAIL is used as a key gene for the treatment of colon cancer. In the field of gene therapy for cancer, this innovative and multifunctional nuclear-targeted nanoparticle was used for the efficient delivery of TRAIL in vivo. When this nanosystem carries the TRAIL gene, it mediates a significant depletion of cancer stem cell-like cells, showing strong therapeutic potential.

This idea was also applied to synthesize RRPHC/pSTA material by Yang et al. [51]. When this material was combined with a therapeutic gene, the apoptin gene engineered with secretary peptide (SP) and transcriptional transactivator (TAT) sequences to obtain SP-TAT-apoptin quality granules (pSTA), compounds could be built to produce membrane-penetrating proteins in large quantities. Thus, once phosphorylated, these cancer-killing proteins rush to the nucleus to achieve cell killing. Experiments demonstrated that RRPHC possesses an excellent transfection effect and a prolonged cellular retention period, and it exerts a strong inhibitory effect on A549 cells (non-small-cell lung cancer cell line). HA carries a large amount of negative charge, and, in blood vessels, this charge on the surface of the material helps to maintain its stability in the bloodstream, and the positive charge is more likely to be phagocytosed by macrophages after adsorption of proteins. PF33 and RRPH contribute to DNA coagulation; biological experiments showed that RRPHC/pSTA can effectively deliver DNA into the nucleus through endosomal/lysosomal escape, laying the foundation for gene transfection. In summary, pSTA-encapsulated RRPHC nanoparticles have potential applications in cancer gene therapy.

### 3.3. Photodynamic Therapy

Photodynamic therapy (PDT) employs organic photosensitizers activated by light to generate cytotoxic reactive oxygen species (ROS), which induce irreversible biomolecular damage through DNA strand breaks and repair enzyme inactivation, ultimately triggering tumor cell death [96]. However, the poor production of ROS, their short lifespan, and the restricted diffusion range constrain their efficacy in killing tumor cells. The cell nucleus is the main storage site of DNA, and DNA is the main target of PDT, so the cell nucleus is considered to be the most effective target to achieve PDT. Emerging nuclear-targeted drug delivery systems address this challenge through rationally designed carriers that enable both precise nuclear localization and controlled photosensitizer release. This spatial-temporal optimization significantly enhances ROS production at the target site while minimizing off-target effects, leading to substantially improved tumor cell eradication. Such nucleus-directed strategies not only amplify PDT efficacy but also reduce collateral damage to healthy tissues, representing a paradigm-shifting approach in precision oncology.

**Figure 10:**
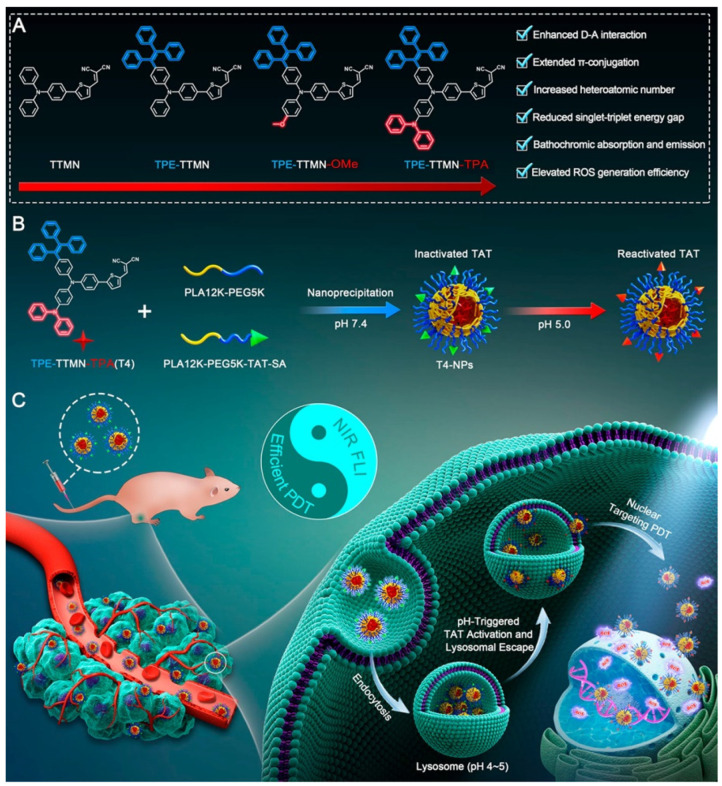
Schematic illustration of (**A**) advanced molecular engineering of high-efficiency AIE-active photosensitizers, (**B**) fabrication of acid-responsive, nuclear-targeting AIE nanotheranostics, and (**C**) utilization in accurate and efficacious photodynamic cancer treatment. Reprinted with permission from [52]. Copyright 2021 American Chemical Society.

Zhang et al. crafted a nanocarrier system containing finely engineered photosensitizers with aggregation-induced emission (AIE) that allowed them to have broad absorption in the infrared light region to generate large amounts of ROS (Figure 10) [52]. It enables nuclear-targeted delivery functions and tumor lysosomal acid activation, providing a new path for precise and efficient phototherapy. Conventional photosensitizers tend to aggregate, producing insufficient ROS due to their susceptibility to intermolecular π-π stacking in vivo due to hydrophobic interactions. The AIE photosensitizer stimulated by near-infrared (NIR) was finely designed to attain high ROS generation efficiency. The highly distorted conformations of the tetraphenylethylene (TPE) and triphenylamine (TPA) fragments in the molecule can effectively prevent fluorescence from bursting out in the aggregated state, which significantly increases the intermolecular distances and reduces the intermolecular π-π interactions. The SA-TAT was constructed by the TAT peptide modified by succinic anhydride, to attach to the surface of this material, where the succinimide remains stable at normal physiological pH but responds rapidly to endosomal/lysosomal acidity. Then the SA-TAT was modified at the end of maleimide end-capped PEG block polylactide (Mal-PEG-PLA) to synthesize the amphiphilic copolymer SA-TAT-PEG-PLA. Using the co-assembly technology of SA, nanocarriers with acid-triggered nuclear-targeting function were successfully prepared to achieve the precise nuclear-targeting effect. The study used quantitative analysis to compare the ROS-generating efficiency of four AIEgen compounds and demonstrated that T4-NPs can effectively generate ROS under light exposure, inducing apoptosis and necrosis in tumor cells, thereby indirectly indicating potential damage to nuclear DNA. The in vitro and in vivo experimental studies confirmed that this phototherapeutic nanomaterial possessed good biocompatibility and demonstrated excellent performance in NIR-guided PDT. This example of a successful phototherapeutic construct will undoubtedly offer valuable insights for the advancement of more efficacious therapeutic agents and is anticipated to facilitate their widespread adoption in potential clinical applications.

AS1411/Ce6/hemin@pHis-PEG (CACH-PEG) NCP is a kind of G-tetrapod-based nanoscale coordination polymer, which was developed by Yang et al. The innovative design of this nanostructure enables the translocation of the photosensitizer Ce6 in the nucleus, allowing ROS to be generated in the nucleus and improving efficacy. Meanwhile, AS1411 can effectively inhibit the expression of the anti-apoptotic protein Bcl-2 and significantly enhance PDT-induced apoptosis. In addition, G-quadruplex and hemin (an iron-containing porphyrin) in these nanomedicine carriers possess DNAzyme functions that mimic catalase, which can decompose H_2_O_2_ within the tumor and generate oxygen in situ, thus overcoming hypoxia-associated therapeutic resistance and further enhancing the efficacy of PDT. Using methods such as SOSG fluorescence detection, DCFH-DA staining, γ-H2AX focal point analysis and TUNEL staining, this study conducted quantitative and qualitative assessments of the ROS production rate and nuclear DNA damage markers induced by CACH-PEG nanostructures under light exposure. Comparing the experimental results across different treatment groups confirms that CACH-PEG nanostructures effectively increase the ROS production rate and induce nuclear DNA damage under light exposure. The experimental results show that a low dose of CACH-PEG NCP can produce highly effective PDT, which not only achieves intranuclear delivery of photosensitizers and down-regulation of anti-apoptotic proteins, but also regulates the unfavorable tumor microenvironment, thus improving the efficacy of tumor therapy [53].

Cheng et al. used chimeric peptide-modified exosomes as naturally sourced nanomembrane vesicles for plasma membrane- and nuclear-targeted photosensitizer delivery and synergistic PDT [54]. A chimeric peptide-engineered exosome (ChiP-Exo) was designed and fabricated, which enables plasma membrane-targeted PDT. Exosomes are superior biocompatible, less immunogenic [97,98,99,100]. and easier to be internalized by cells [101]. The multifunctional chimeric peptide (ChiP) of C16-K(PpIX)-PKKKRKV incorporates a C16-K alkyl chain for exosome engineering, a PpIX photosensitizer for PDT, and an NLS peptide for nuclear translocation. After careful modification of endogenous exosomes by ChiP, the resulting ChiP-Exo not only exhibits excellent biocompatibility, but also dual targeting of the plasma membrane and nucleus. Leveraging the PDT properties of the plasma membrane targeting of the material, it can directly attack and compromise the membrane structure, thereby inducing cell death. Not only is the material biocompatible, but it also has an enhanced ability to generate ROS. When light is irradiated to the plasma membrane-targeted ChiP-Exo, it effectively activates ROS on-site, disrupting the plasma membrane structure and ultimately causing cell death. The photochemical internalization (PCI) effect and the lysosomal escape mechanism worked together to optimize the delivery efficiency of ChiP-Exo to the cytoplasm during the first phase of light exposure. Subsequently, the NLS peptide conferred the nuclear-targeting ability to ChiP-Exo, which further enhanced the intranuclear PDT effect under second-stage light exposure. Using methods such as fluorescence spectroscopy, intracellular ROS detection, TUNEL staining, H&E staining, and analyses of cell viability and apoptosis, it was demonstrated that ChiP-Exo effectively generates ROS under light exposure, leading to significant nuclear DNA damage and cell apoptosis. Validated by in vitro and in vivo studies, the subcellular dual-targeting strategy of ChiP-Exo demonstrated significant tumor suppression under dual-phase light exposure.

### 3.4. Photothermal Therapy

Photothermal therapy (PTT) utilizes near-infrared (NIR)-responsive photothermal agents (PTAs) to achieve localized hyperthermia for tumor ablation, offering distinct advantages in non-invasiveness, tissue specificity, and minimized systemic toxicity. PTT has demonstrated its unique appeal with its less invasiveness, mild side effects and high specificity [102,103]. Targeting the photosensitizer to the nucleus of the tumor cells enables the exact focusing of the therapeutic energy to achieve precise targeting of the tumor cells. This can not only effectively improve the therapeutic effect and reduce the damage of normal cells, but also reduce the side effects during the treatment process. Therefore, the construction of a nuclear-targeted drug delivery system is an important research direction of photothermal therapy and has important research significance.

Li et al. constructed cancer cell nuclear-targeted copper sulfide nanoparticles, CuS@MSN-TAT-RGD [55], with significant photothermal effects, which can utilize the electronic energy level transition within copper nanoparticles (Cu NPs) to emit energy, thereby elevating the temperature. Cu NPs are considered to be the optimal core material [104,105], and efficient uptake of the nucleus was achieved by encapsulating Cu NPs in MSNs and modifying them with TAT peptides. Meanwhile, to further increase the precision of treatment, RGD peptide was used for modification. Under 980 nm laser light, the heat generated by these nanoparticles can significantly raise the temperature inside the cell nucleus, which in turn triggers DNA damage and protein denaturation, thus killing the cancer cells and preventing cancer recurrence. The in vivo experiments showed that the transplanted tumor was completely cleared after 14 days with no signs of recurrence after just one treatment. This breakthrough not only fully verifies the excellent therapeutic effect of the designed nanoparticles, but also opens up new avenues for future tumor therapy and shows great potential for application.

**Figure 11:**
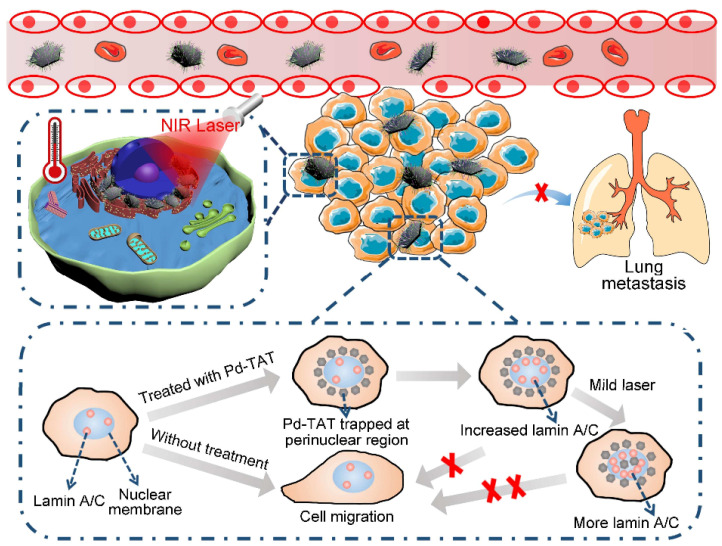
Schematic illustrating the synthesis process of Pd-TAT nanoparticles, designed for perinuclear targeting and capable of inhibiting cancer cell migration as well as photothermal therapy. Reprinted with permission from [56]. Copyright 2019 Elsevier.

Gao and his team designed a similar material by modifying TAT peptides on palladium nanosheets (Pd NSs) to form Pd-TAT (Figure 11) [56]. Two-dimensional palladium nanosheets have strong light absorption in the near-infrared region and have a photothermal conversion efficiency of up to 52.0% with high photothermal stability [106,107]. Pd-TAT also has the ability to stimulate the overexpression of lamin A/C proteins, which correlates with the nucleus’ mechanical stiffness. This mechanism heightens the mechanical stiffness of the nucleus, thereby significantly effectively slowing down the migration of cancer cells. Under low-power (0.3 W/cm^2^) laser irradiation, localized thermotherapy in the nuclear periphery is generated, which leads to increased permeability of the nuclear membrane and more Pd-TAT into the nuclear region, increasing nuclear stiffness. Such increased permeability creates favorable conditions for DNA-damaging agents (e.g., chemotherapeutic drugs) to overcome the nuclear barrier and efficiently act on intranuclear DNA targets. Furthermore, lamin A/C modulation may interfere with DNA repair pathways (e.g., homologous recombination repair) or promote apoptosis-related signaling, further potentiating tumor cell death induced by DNA-damaging agents. Collectively, these mechanisms underpin the synergistic antitumor effect of “hyperthermia + DNA-damaging agents” and provide new insights into the deeper mechanisms of nuclear-targeted therapy. The material is capable of effectively modulating the migration rate of cancer cells and can induce tumor ablation through high-temperature effects.

**Figure 12:**
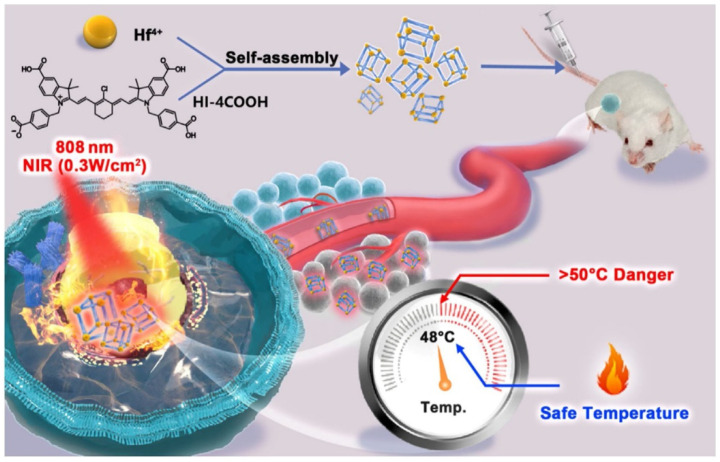
Fabrication of Hf-HI-4COOH Core–Shell Nanostructures and Exploration of Their Application in Nuclear-Targeted Low-Temperature PTT. Reprinted with permission from [57]. Copyright 2020 Elsevier.

Jiang and his team have reported a novel nanoscale coordination polymer (NCP) based on heptamethine cyanine dye, named Hf-HI-4COOH (Figure 12) [57]. This was a low-temperature nuclear-targeted PTT photosensitizer (PS), and the lower temperature (<50 °C) and low power (<0.4 W/cm^2^) reduced the damage to the skin and normal organs in PTT treatment [108]. The strong bond between Hf and the carboxyl group allowed Hf to cooperate with HI-4COOH to form a robust NCP structure of suitable size. The dye molecules were effectively dispersed in this material, effectively avoiding the aggregation and self-burst phenomena that may occur under NIR irradiation. Meanwhile, the heptamethine cyanine dye ligand significantly enhanced the photothermal conversion efficiency through the coordination of its carboxylate to the heavy Hf center. These properties endowed PS with excellent nuclear-targeting due to its specific particle size and the positive charge carried on its surface. In cellular localization experiments, the materials were able to effectively target the nucleus of cells observed, and demonstrated significant therapeutic effects in phototherapy experiments, thereby providing further validation of their nuclear-targeting capabilities. In the in vivo mouse efficacy experiments, mice treated with Hf-HI-4COOH showed significant tumor shrinkage and even achieved complete eradication of tumors at lower power densities and temperatures. Throughout the treatment process, the mice did not show obvious toxic reactions, which proved the safety of Hf-HI-4COOH. In addition, after 14 days of observation, Hf-HI-4COOH was effectively cleared in mice, and the blood analysis results showed no obvious changes. Nuclear-targeted Hf-HI-4COOH, as a novel PS, provides strong support for safe PTT at low power density and low temperature. Its nuclear-targeting property makes the treatment more precise and ensures the safety of the treatment process, opening up a new path for the development of new-generation PTT technology.

### 3.5. Sonodynamic Therapy

Sonodynamic therapy (SDT) has emerged as a promising non-invasive modality for deep-seated tumors [109], leveraging ultrasound-activated sonosensitizers to generate cytotoxic reactive oxygen species (ROS) via acoustic cavitation effects [101]. SDT is characterized by its non-invasive and exceptional tissue penetration capacity [102]. Spatial confinement of ROS production within nuclei induces direct DNA double-strand breaks, such subcellular precision not only enhances therapeutic efficacy but reduces off-target toxicity to adjacent normal cells, representing a transformative strategy to overcome the depth limitations of optical therapies.

**Figure 13:**
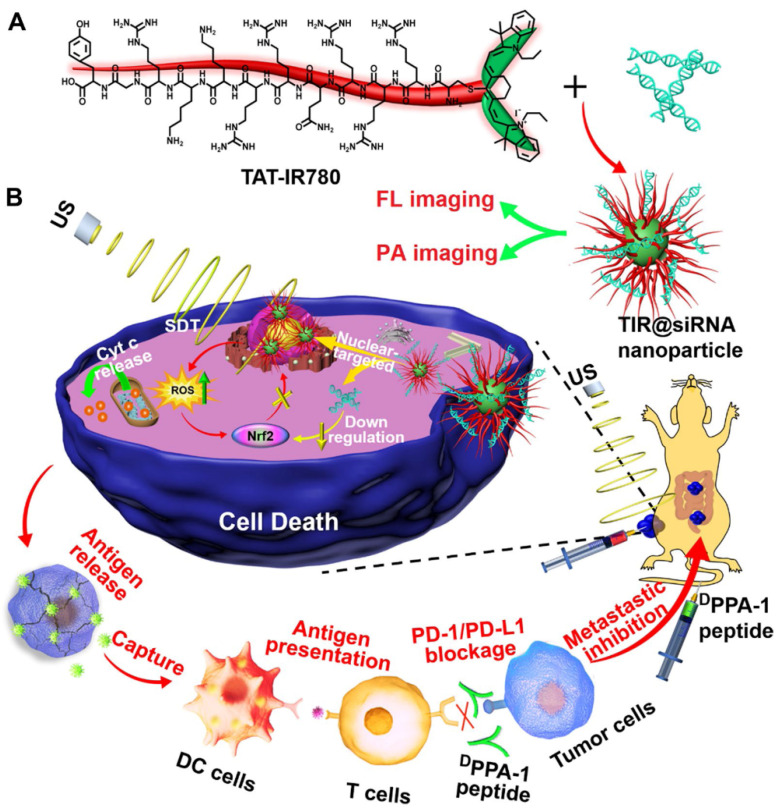
Schematic illustration of TIR@siRNA. (**A**) Preparation of TIR@siRNA nanoparticles, and (**B**) their functional mechanisms in combating colorectal cancer are elucidated through gene-enhanced, nuclear-targeted SDT, which potentiates anti-PD-L1 therapy both in vitro and in vivo. Reprinted with permission from [58]. Copyright 2021 Biomed Central Ltd.

Wan et al. designed a nuclear-targeted photosensitizer [58], TATIR780 (TIR), to load Nrf2-siRNA, which was self-assembled by electrostatic interaction to form solid nanospheres of TIR@siRNA with a particle size of about 5 nm (Figure 13). This structure enhances the nuclear-targeted SDT and further promotes the peptide-based anti-PDL1 therapy, which provides a new avenue for the treatment of colon cancer. IR780 is an acoustic sensitizer that is widely used in the treatment of SDT [110,111]. Its modification by TAT peptide not only served the nuclear-targeting function but also increased the hydrophilicity of IR780. Nrf2-siRNA effectively inhibited the up-regulated expression of Nrf2 after SDT treatment by stabilizing the maintenance of high levels of intracellular ROS, thereby blocking the regulatory pathway of redox homeostasis, which in turn significantly enhanced the TIR-mediated effects of SDT in anti-tumor. Its modification by TAT peptide not only served the nuclear-targeting function, but also increased the hydrophilicity of IR780. Nrf2-siRNA effectively inhibited the up-regulated expression of Nrf2 after SDT treatment by stabilizing the maintenance of high levels of intracellular ROS, thereby blocking the regulatory pathway of redox homeostasis, which in turn significantly enhanced the TIR-mediated effects of SDT in anti-tumor. Furthermore, this gene-enhanced nuclear-targeted SDT strategy not only induces ICD and improves the immunosuppressive microenvironment, but also promotes the development of anti-pd-L1 therapy based on DPPA-1 peptide and effectively inhibits intestinal metastasis of colorectal cancer by activating systemic immune responses, providing a new strategy for tumor therapy.

Zhang and colleagues have developed a nuclear-targeted Ti-tetrakis(4-carboxyphenyl)porphyrin (TCPP) metal–organic framework (MOF) platform [59], which acts as an acoustic sensitizer for direct and effective nuclear-targeting to promote more effective SDT due to its small size (intracellular < 10 nm) and charge reversal-enabling properties. The creation of this MOF platform paves the way for oxygen-independent SDT, offering novel treatment options for pancreatic cancer and innovative approaches for addressing hypoxic tumors.

### 3.6. Synergistic Therapy

Synergistic treatment of tumors is an innovative, multifaceted therapeutic strategy that combines various treatments to maximize the benefits of each treatment while minimizing side effects. Synergistic therapy allows for the development of personalized treatment plans and precise adjustments, offering an ideal solution for addressing the complexity of tumor heterogeneity. In synergistic therapy, the nucleus of the cell, as the life center of the cell, is a key link in achieving precision therapy. By directing drugs or therapeutic agents with high precision to the cell nucleus, directly attacking the genetic material of tumor cells could be achieved. This approach can destroy their ability to grow and divide and enhance the lethality of tumor cells, as well as reduce the damage to normal cells and reduce side effects during treatment. Nucleus targeting plays an active role in synergistic tumor therapy, which can bring better treatment experiences to patients and improve survival rates.

**Figure 14:**
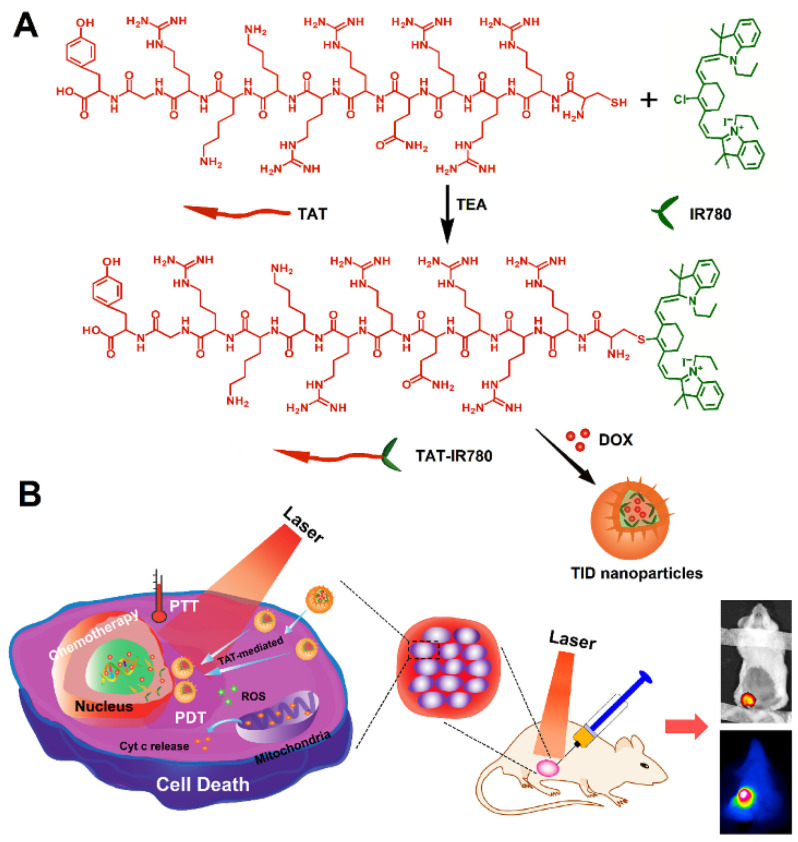
Schematic illustration of TIR@siRNA. (**A**). The synthesis of TAT-IR780 and preparation of TID NPs, and (**B**). synergistic effects of TID nanoparticles-mediated trimodal treatment of nuclear-targeted PTT/PDT and chemotherapy against breast cancer. Reprinted with permission from [60]. Copyright 2020 Elsevier.

Wan et al. developed a nuclear-targeted delivery system combining PTT/PDT and chemotherapy for the treatment of breast cancer [60], in which a near-infrared fluorescent dye, IR780, was coupled with a TAT peptide to obtain a conjugated system, TAT-IR780, which is hydrophilic and photosensitized, and then DOX was encapsulated within the TAT-IR780 by self-assembly to form TID particles with a particle size of approximately 100 nm (Figure 14). The TAT peptide mediated the entry of TID nanoparticles into the nucleus and significantly improved the internalization of IR780. Experiments have shown that under 785 nm laser irradiation, TID nanoparticles rapidly destroy the genetic material of breast cancer cells, effectively driving these cells toward apoptosis. When TID nanoparticles were injected inside the tumors of mice with breast cancer and combined with local laser irradiation, not only the precise guidance of fluorescence and photothermal imaging was achieved, but also the combination of nuclear-targeted PTT/PDT and chemotherapy treatment protocol. This strategy demonstrated significant synergistic effects on breast tumor ablation and recurrence control, providing an innovative nuclear-targeted nano-platform for photochemotherapy that integrates dual imaging guidance to significantly enhance the precision and efficacy of treatment.

An emerging oncological treatment strategy combining chemotherapy and chemodynamic therapy (CDT) was crafted by Xin et al. [61], and they developed a new carrier-free nanomedicine (CMC-DD2) (Figure 14). This drug combines a natural melanin complex with excellent CDT properties with a dehydroconiferylate hexamer (DD2) with potent anti-tumor activity by self-assembly. This combination is designed to achieve synergy between chemotherapy and CDT, providing a new strategy for tumor treatment. Chitosan-melanin complex (CMC) is a biologically active amphiphilic nuclear-targeting substance with good chemotherapeutic properties that produces dOH in water and is an excellent catalyst for CDT. CMC shows excellent properties in pH-responsive charge reversal and lysosomal escape, which contributes to its long circulation in the bloodstream, facilitating cellular uptake and drug transport [112,113], thus enhancing therapeutic effectiveness. Dehydrofolate hexamer (DD2) is a natural and highly effective chemotherapeutic agent that is not only hydrophobic but also has a low adverse reaction rate [114], and its unique strongly hydrophobic aromatic tricyclic structure makes it an ideal ‘active’ core for nanomedicines. The pH-responsive property of CMC-DD2 was exploited to achieve charge flipping in tumor tissues, thus making it easier to be internalized by cells. After the lysosomal escape, the drug can accumulate in the nucleus and bind directly to DNA, thereby changing its conformation. In the presence of intracellular reactive oxygen species (ROS), the nanomedicine dissociates, releasing dOH and DD2, which together act on the DNA to cause severe damage, resulting in efficient tumor therapy.

**Figure 15:**
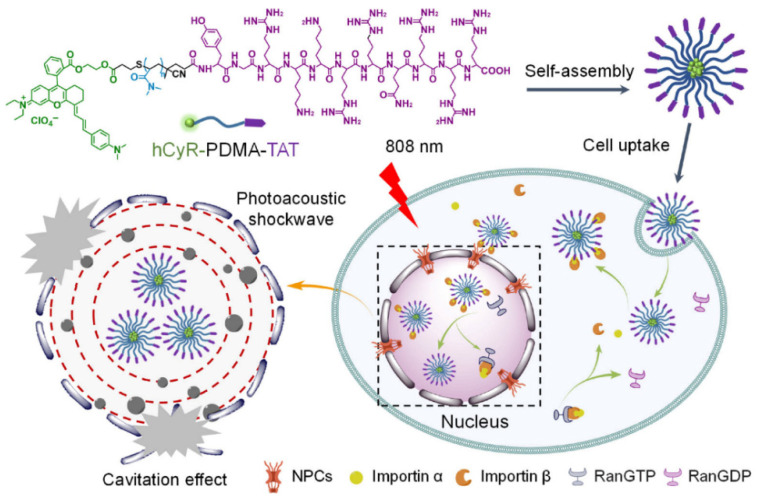
Schematic illustration of TAT peptide-conjugated nanoparticles for nucleus-specific cavitation in photoacoustic cancer therapy. Reprinted with permission from [62]. Copyright 2020 Springer Nature.

Wang et al. developed a photoacoustic therapeutic polymer that enables efficient nuclear-targeting for fluorescence and photoacoustic dual-mode imaging (Figure 15) [62]. Photoacoustic therapy is an emerging technology in cancer-specific therapeutic techniques that offers unique advantages to compensate for the shortcomings of traditional treatment methods [115,116]. This innovative therapy not only significantly improves the accuracy of treatment, but also dramatically reduces the potential damage to normal tissues [117,118]. When the light absorber is irradiated by the pulsed laser beam, the light energy is rapidly converted into acoustic energy, forming a powerful photoacoustic shock wave [119,120]. This shock wave then triggers a cavitation effect, which acts directly on the tumor cells to achieve effective destruction. Photoacoustic therapy has injected new vitality into the field of cancer treatment with its precise, efficient and safe features. They achieved precise nuclear-targeting by linking TAT peptides, hydrophilic groups (N,N-dimethylacrylamide) (PDMA), and near-infrared (NIR) light absorbers (hCyR), which were self-assembled into particles with a particle size of about 28 nm (TAT-PDMA-hCyR NPs). The material can precisely damage the nucleus of tumor cells when irradiated by 808 nm laser, thus triggering the cell death mechanism and achieving efficient anti-tumor therapeutic effects.

## 4. Tumor Targeting of Other Subcellular Structures

In addition to the nucleus, therapeutic agents also affect a number of other important subcellular organelles for the purpose of tumor therapy. In this section, the role of key targeted organelles in tumor therapy will be briefly described.

Mitochondria are the energy factories of an organism and provide ATP for various cellular activities within the cell. Apoptosis can be induced in tumor therapy by disrupting the energy metabolic pathways of tumor cells [121]. Shen et al. developed gold nanorods (AuNRs) with specific targeting peptides that can be used as biocompatible organelle-targeting nanoprobes [122]. These nanoprobes enable direct analysis of subcellular biomolecules and important organelles using surface enhanced Raman scattering (SERS) spectroscopy. In addition, these organelle-targeting AuNRs have been applied to the photothermal treatment of a variety of cancer cells, including HepG2, HeLa and MCF-7 cell lines. Cell viability assay experiments showed that AuNRs targeting nuclei and mitochondria exhibited higher photothermal efficiency under 808 nm laser irradiation compared to lysosome-targeted AuNRs.

As a key regulatory center in the cell, the lysosome plays an important role in directing and controlling cellular metabolism and signaling. It is closely linked to cellular survival and is involved in cell fate decision-making processes such as apoptosis, necrosis and autophagy [123,124,125] and is indispensable for the maintenance of cellular homeostasis. As the terminal point of cellular degradation, the lysosome carries multiple missions. Its interior is rich in various hydrolytic enzymes, which not only regulate cellular metabolic activities, but also deeply participate in a variety of physiological processes. It plays an important role in key aspects such as cell signaling and cell death [126], demonstrating its powerful function as an intracellular regulatory center. To efficiently direct small molecules or metal complexes into the lysosome, the strategy of coupling therapeutic agents to alkaline targeting units is often employed. In molecular targeting of lysosomes, compounds such as 4-(2-hydroxyethyl)lysine, N, N-dimethylethylenediamine, and diethylene glycol methyl ether are often used as effective targeting fractions for precise drug delivery and release [127]. Peng et al. have crafted a novel ruthenium complex (Ru-I) tailored for red-light-activated photodynamic therapy (PDT) [128]. This photosensitizer has a positive surface charge and enhanced water solubility, which can be rapidly internalized by the cell and accumulated in the lysosome. Upon irradiation with infrared light, Ru-I efficiently produces reactive oxygen species, culminating in lysosomal disruption and subsequent cell apoptosis. The results suggest that this red-light-responsive Ru complex photosensitizer has potential applications for lysosome-localized photodynamic therapy for the treatment of diseases such as cancer. As the first PDT photosensitizer suitable for lysosomal targeting, Ru-I brings fresh hope to the field of anti-cancer research. This innovation not only expands the application scope of PDT but also provides a brand new opportunity for lysosomal-targeted PDT, heralding a new chapter in future cancer treatment strategies.

The endoplasmic reticulum is a continuous and massive membrane system and is the largest organelle in eukaryotic cells. Its core responsibilities lie in the synthesis, folding, and post-translational modification of cellular secreted and transmembrane proteins, as well as the transport of these proteins within the cell [129]. The endoplasmic reticulum, with its fine membrane structure and efficient substance transport capacity, ensures that proteins are properly processed and precisely transported within the cell, thus maintaining normal cellular function. When the cell is stimulated, the endoplasmic reticulum is stressed to disrupt the redox balance of the cell [125]. Tumor growth and development are associated with endoplasmic reticulum stress [130]. Severe endoplasmic reticulum stress continuously impairs the regulatory functions of the endoplasmic reticulum as well as other subcellular organelles, not only interfering with autophagy processes, but also affecting the reprogramming and adaptive capacity of cells. More seriously, this stressful state may trigger apoptotic mechanisms, thereby inducing tumor cell death [131]. Zhou et al. have developed a metal complex, Ir-Rho-G2, which exhibits excellent reactive oxygen species generation efficiency and highly specific endoplasmic reticulum localization [132]. In addition, the complex has low dark cytotoxicity, which ensures its safety in the cellular environment; its high photostability allows the complex to maintain stable performance in complex biological environments, and, more importantly, it has selective tumor cell uptake, which provides the possibility of precise targeting for tumor therapy.

## 5. Conclusions and Outlook

With the ongoing advancements and technological innovations in the fields of biomedicine and materials science, the development of nuclear-targeted nanomaterials has emerged as a central focus in cancer therapy and holds significant promise for future development. The application of nanotechnology in treatment can directly transport drugs to the site of action, thereby maximizing therapeutic efficacy and reducing drug side effects. Although nuclear-targeting strategies have numerous advantages, it is undeniable that they also have some challenges and limitations, particularly in clinical translation.

1. Tumor heterogeneity contributes to considerable variability in treatment outcomes among individuals. Consequently, it is essential to investigate the roles of various cellular signaling pathways and molecular regulators in tumors, to reveal the underlying intrinsic molecular regulatory mechanisms and identify novel molecular targets, which are crucial for achieving organelle-targeted therapy for most cancers. This will facilitate the development of novel delivery strategies, identification of additional therapeutic targets, and enhancement of treatment efficacy.
2. The safety of targeted therapies remains inadequately investigated. Metallic nanoparticles provide a range of strategies for tumor therapy, enabling the targeted delivery of drugs to the nucleus while inhibiting DNA repair mechanisms. However, there are undeniable concerns about their safety. Due to their particle size and biodistribution, the nanoparticles may cause severe visceral inflammation in the organism [133]. The role of targeting peptides is equally important and significant in the construction of nuclear-targeted nanocarriers. Among them, the TAT peptide, one of the most commonly used nuclear-targeting peptides, has been widely employed, though several studies have revealed its potential risk of neurotoxicity [134,135]. In addition, TAT peptide-conjugated drugs may also undergo retrograde delivery when released in the cytoplasm before their intended release, potentially leading to the accumulation of the drug at other non-target sites, which may raise further safety concerns [136,137].
3. The delivery efficiency and spatiotemporal control of nuclear-targeted nanodrugs are still the core bottlenecks in current research. Although TAT peptide-modified systems can increase the intranuclear accumulation rate through active transport mediated by nuclear localization signals, it is still far below the threshold of drug concentration required for therapy. Meanwhile, the spatial and temporal precision of drug release in existing systems is insufficient, resulting in a significant reduction in effective drugs reaching the nucleus.
4. Nuclear-targeting nanocarriers hold immense potential for revolutionizing cancer treatment by delivering therapeutic agents precisely to the cell nucleus, and their prospects for clinical translation are highly promising. However, one of the primary challenges is achieving large-scale production while ensuring the consistency of the nanocarriers’ quality and performance. In a laboratory setting, these systems are typically synthesized in small batches using optimized protocols. Scaling up production, however, introduces numerous complexities, such as variations in reaction conditions, raw material quality, and equipment performance. These variations may lead to batch-to-batch inconsistencies in the size, shape, surface charge, and drug-loading capacity of the nanocarriers, thereby compromising their targeting efficiency and therapeutic efficacy. Furthermore, the toxicity and long-term safety of the constituent nanomaterials are of paramount importance. Although they may possess excellent drug delivery properties, their complex interactions with biological systems can be difficult to predict, potentially leading to adverse reactions. The regulatory landscape for these advanced therapeutics is also complex and evolving. Unlike conventional small-molecule drugs, nanocarriers are regarded as combination products, integrating both the active pharmaceutical ingredient and the delivery vehicle. This dual nature presents challenges in terms of regulatory classification, approval pathways, and quality control standards. Moreover, global regulatory bodies often have divergent requirements for evaluating nanomedicines. The approval process is exacerbated by the lack of standardized methods for characterizing nanomaterials and assessing their safety and efficacy. Ultimately, establishing a clear and unified regulatory framework is crucial for accelerating the clinical translation of nuclear-targeting nanocarriers.

In the future, cell nuclear-targeting technology may be integrated with other treatment modalities to form a more integrated and personalized treatment plan to provide more effective treatment for patients. At the same time, it is worthwhile to explore more perfect cytosolic targeting technology and promote its application in the field of tumor therapy, which deserves more in-depth research. Future research should prioritize the following directions:

1. Non-peptide nuclear-targeting ligands: Current strategies heavily rely on TAT peptide-mediated nuclear transport, yet its neurotoxicity and retrograde cytoplasmic release raises safety concerns. Developing non-peptide ligands (e.g., small molecules or synthetic polymers) with comparable targeting efficiency but reduced immunogenicity is imperative.
2. Dynamic nuclear barrier modulation: While direct nuclear membrane disruption via light or ROS shows promise, optimizing spatiotemporal control to minimize off-target damage remains challenging. Integrating stimuli-responsive materials (e.g., pH/redox/enzyme-triggered systems) could enhance precision.
3. Multimodal synergy validation: Combining nuclear-targeted delivery with emerging modalities (e.g., CRISPR-based gene editing or radiopharmaceuticals) requires systematic evaluation to address tumor heterogeneity. Standardized preclinical models mimicking human nuclear transport dynamics are needed for translational validation.

In summary, nuclear-targeted DDS has emerged as a transformative paradigm in oncology by directly addressing the critical bottleneck of subcellular drug localization. Current strategies leveraging NPC permeability modulation, ligand-mediated active transport, and stimuli-responsive nanocarriers have achieved improvements in intranuclear drug accumulation compared to conventional chemotherapy, translating reductions in effective doses across preclinical models. With the continuous development and progress of science and technology, more accurate and efficient nuclear-targeting strategies are expected to be developed to achieve precise killing of tumor cells and reduce the generation of toxic side effects. In the future, cell nuclear-targeting technology may be integrated with other treatment modalities to form a more integrated and personalized treatment plan to provide more effective treatment for patients. At the same time, it is worthwhile to explore more perfect cytosolic targeting technology and promote its application in the field of tumor therapy, which deserves more in-depth research.

## Figures and Tables

**Table 1:** Summary of various nanoparticles capable of targeting the cell nucleus.

Nanoparticles	Sizes	Release Kinetics	Nuclear-Targeting Mechanism	Subjects	In Vivo Tumor Inhibition Rates	Reference
aPD-L1 and ZnPc be delivered by graphene quantum dots (GQDs)	5 nm	Laser	Passive diffusion	Human OSCC cell lines SCC9/SCC25 and HOKs; Female C3H mice	~80%	[35]
a 10-hydroxycamptothecin—peptide conjugate HpYss	50~100 nm, can be reformatted to nanofibers (4~9 nm)	GSH	Passive diffusion	HepG2 cells; Female BALB/c nude mice	81%	[36]
poly(ethylene glycol)-poly(ε-caprolactone) with 2,3-dimethylmaleic anhydride-Tat decoration	82.1 ± 5.14 nm	pH	Active targeting via CPPs	Mouse fibroblast L929 cells; human non-small-cell lung cancer A549 cells, and mouse breast cancer 4T1 cells; Balb/c female mice	90%	[37]
Intelligent biodegradable hollow HMnO2 nanoparticles with a human umbilical cord mesenchymal stem cell membrane coating, loading with a TAT peptide-modified membrane structure	116.5 ± 5.7 nm	pH	Active targeting via CPPs	LO2 cells, BEAS-2B cells, A549 cells; BALB/c nude mice	85%	[38]
Dynamic PEGylation of a borate-based coordination polymer-coated PDA nanoparticle	242 ± 13.7 nm	pH	Active targeting via phenylboronic acid	MCF-7 cells; nude mice	96%	[39]
HKRK nanoparticle-stabilized capsule platform to deliver benzyl boronate-modified proteins	112 kD	pH	Active targeting via phenylboronic acid	HeLa cell	—	[40]
An artificial metalloenzyme with copper cluster firmly anchored in bovine serum albumin conjugated with tumor-targeting peptide	4.9~6.6 nm	redox	Active targeting via metal complexes	A549 cells; Female BABL/c athymic nude mice	67%	[41]
The NLCs decorated with a polycationic CPP, and coated with polyphosphates	146~152 nm	charge Reversal	Active targeting via charge Reversal	Caco-2 cells	—	[42]
Au NPs	—	Photothermal	Open the Nuclear Pore	HeLa cell	—	[43]
An nanoparticle consisted of a polyamine unit polyhedral oligomeric silsesquioxane, a hydrophilic polyethylene glycol chain, and a photosensitizer rose Bengal	75 nm	Laser	Open the Nuclear Pore	A549 cells	—	[44]
A hierarchical responsive nanomedicine	85.6 ± 11.9 nm	GSH	Active targeting via charge Reversal	U87MG cells; U87MG-tumor-bearing mice	~82%	[45]
(Low molecular weight heparin) LMHP-DOX nanoparticles	39.87 ± 7.23 nm	pH	Active targeting via Histone H1	PC-3M cells; Male Sprague-Dawley (SD) rats and female BALB/C nude mice	~93%	[46]
A mesoporous silica nanoparticle (MSN)-based nuclear-targeted system	91.6 nm	Photothermal	Active targeting via CPPs	MCF-7 cells; BALB/c nude mice	96%	[47]
A novel 1D semiconducting zinc coordination polymer coating with polyacrylic acid to load CPT	115 nm	pH	Active targeting via Zn-O coordination	4T1, 293T or HeLa cells; BALB/c female mice	50%	[48]
An integrin-targeted, cell-permeable and nucleocytoplasmic trafficking peptide-conjugated AIEgen	76~198 nm	enzymes	Active targeting via CPPs	MDA-MB-231 cells; the tumor- bearing mice	—	[49]
PF33/pDNA encapsulated with capsid-like shell RGD-R8-PEG-HA	132.8 ± 3.8 nm	pH	Active targeting via CPPs	HCT116 cells; e female BALB/C nude mice	~75%	[50]
Fluorinated branched polyethylenimine (PF) and RGD-R8-PEG-HA (RRPH), mixed with plasmids	270.0 ± 12.1 nm	pH	Active targeting via CPPs	A549 cells and 293T cells; Balb/c nude mice	~89%	[51]
Acid-Activated Nuclear-targeted AIE Nanotheranostics	50~70 nm	pH	Active targeting via CPPs	4T1 cells; BALB/c nude mice	78%	[52]
Ca-AS1411/Ce6/hemin@pHis-PEG (CACH-PEG) NCP	70 nm (pH = 7.4) and 65 nm (pH = 6.5)	pH	Active targeting via CPPs	4T1 cells; Balb/c mice	~80%	[53]
Exosomes engineered by chimeric peptide	114.0 nm	Laser	Active targeting via CPPs	4T1 cells; CT26 cells; HeLa cells; B16 cells; COS7 cells; female BALB/c mice	~83%	[54]
A cancer cell nuclear-targeted copper sulfide nanoparticles	65 nm	laser	Active targeting via CPPs	HeLa cells; nude mice	100%	[55]
TAT-modified Pd NSs	an average diameter of 19.7 ± 1.9 nm and thickness of 1.9 ± 0.4 nm	laser	Active targeting via CPPs	4T1 cells; U14 cells; MCF-7 cells; nude mice	100%	[56]
A heptamethine cyanine dye-based nanoscale coordination polymer	36.7 nm	laser	Active targeting via metal complexes	4T1 cells; 4T1 tumor-bearing mice	100%	[57]
A nuclear-targeting sonosensitizer based on the TAT peptide coupled IR780 (TAT-IR780) to load Nrf2-siRNA	56.45 nm	ultrasound	Active targeting via CPPs	CT26 cells; Female BALB/c mice	100%	[58]
A nuclear-targeted ultra-small Ti-tetrakis(4-carboxyphenyl)porphyrin metal–organic framework	12.21 ± 1.27 nm	pH; laser	Active targeting via metal complexes	BxPC-3 cells; Female nude mice	78%	[59]
A nuclear-targeting photosensitizer by conjugation of IR780 with TAT peptide to load DOX	100 nm	pH; laser	Active targeting via CPPs	4T1 cells; Female BALB/c mice	~90%	[60]
A carrier-free, nuclear-targeting nanodrug	40 nm	pH; redox	Active targeting via a natural bioactive material	HepG2 cells; HL-7702 cells; Male BALB/c nude mice	~80%	[61]
A nuclear-targeted photoacoustic theranostic polymer	28 nm	Enzymes; laser	Active targeting via CPPs	EMT6 cells; BALB/c mice	~90%	[62]

## Data Availability

Data are available on request.
